# Stair-descent phenotypes in community-dwelling older adults determined using high-level balance tasks

**DOI:** 10.1007/s40520-025-02929-5

**Published:** 2025-01-29

**Authors:** Takahiro Tanaka, Kimitaka Hase, Kimihiko Mori, Masanori Wakida, Yasuaki Arima, Takanari Kubo, Meguru Taguchi

**Affiliations:** 1 Department of Physical Medicine and Rehabilitation, Kansai Medical University, Osaka, Japan; 2 Department of Physical Therapy, Aino University, 4-5-4 Higashioda, Ibaraki, Osaka, 567-0012 Japan; 3 Department of Rehabilitation, Kansai Medical University Hospital, Osaka, Japan; 4 Department of Physical Therapy, Faculty of Rehabilitation, Kansai Medical University, Osaka, Japan

**Keywords:** Older adults, Stair descent, Clustering, Fall risk, Physical function

## Abstract

**Background:**

Falls on stairs are a major cause of severe injuries among older adults, with stair descent posing significantly greater risks than ascent. Variations in stair descent phenotypes may reflect differences in physical function and biomechanical stability, and their identification may prevent falls.

**Aims:**

This study aims to classify stair descent phenotypes in older adults and investigate the biomechanical and physical functional differences between these phenotypes using hierarchical cluster analysis.

**Methods:**

Eighty-two older adults participated in this study. Stair descent was measured using a three-dimensional motion analysis system. Physical function was assessed using measures of muscle strength, walking speed, the Timed Up and Go Test (TUG), and the Community Balance and Mobility Scale (CB&M).

**Results:**

Hierarchical cluster analysis was performed on kinematic data obtained during stair descent. Three phenotypes were identified: neutral (N-type; 24%), extension (E-type; 52%), and rotation (R-type; 23%). There were no significant differences in lower limb muscle strength or walking speed among the different types, and TUG scores showed no differences in terms of mobility or balance abilities. However, CB&M scores were significantly lower for E-type and R-type compared to N-type. Sub-analyses revealed that while there were no differences in the mobility factor of CB&M between E-type and R-type, the strength factors were significantly lower compared to those for N-type.

**Discussion:**

These results suggest that E-type and R-type stair-descent patterns may be influenced by declines in standing balance ability and muscle strength.

**Conclusions:**

These findings may inform fall-prevention training programs related to stair descent among older adults.

## Introduction

Stairs are one of the most common sites for fall-related accidents among older adults [1, 2]; stair falls cause more serious injuries that may eventually be fatal than falls on level ground [3]. Since fall-related mortality rates have increased significantly among those aged 65 and over in many countries [2, 4, 5], fall prevention is necessary on both level ground and stairs. Fall-prevention measures are more important during stair descent, as descent is three times more dangerous than ascent [6].

Clarifying the biomechanical features of stair descent among older adults may contribute to fall prevention. Several biomechanical studies show features of stair descent among older adults compared with young adults: smaller maximum knee flexion angle during descent [7, 8], and lower ankle plantar flexion moment. Knee extension moments are the same or lower [7]. Furthermore, older adults’ center of mass (CoM) during descent has a fast anterior excursion velocity and large anterior displacement compared to young adults [7].

These biomechanical features of older adults’ stair descent are not uniform, and there are various types, similar to their walking [9, 10]. Cluster analysis proves to be a valuable approach for categorizing the phenotypes associated with posture and movement patterns. By incorporating kinematic data obtained from motion analysis, cluster analysis facilitates the classification of gait and postural abnormalities, such as knee osteoarthritis [11], hip osteoarthritis [12], and hemiplegia [13]. Since differences in phenotypes are influenced by variations in physical function, classifying movements through clustering and comparing physical functions—such as muscle strength and balance—across clusters may contribute to the development of targeted treatment strategies [12]. However, the relationship between stair descent phenotypes and physical function in older adults has not yet been clarified [9].

Therefore, we hypothesize that the stair descent of older adults can be classified into distinct phenotypes using kinematic data obtained from three-dimensional (3D) motion analysis. Specifically, these phenotypes are likely to include not only the two types previously observed visually—descending with a sideways orientation and descending with trunk instability [5]—but also other patterns resembling those of young adults. We further hypothesize that these phenotypes are influenced by psychological and physical factors, such as fear of falling, muscle strength, and balance ability [5, 14], and each phenotype exhibits unique biomechanical characteristics and physical functional profiles.

This study aims to classify stair-descent phenotypes among community-dwelling older adults using hierarchical cluster analysis, clarifying the differences in biomechanical characteristics and physical function among them.

## Methods

### Participants

To determine the sample size for this study, we referenced prior research guidelines for hierarchical cluster analysis, which suggest a sample size of N = 20 to N = 30 per subgroup to achieve sufficient statistical power [15]. Based on our hypothesis that stair-descent motions can be classified into 2–3 distinct phenotypes, we calculated an optimal sample size of 60 to 90 participants to ensure robust subgroup identification. This range allows for reliable statistical modeling and subgroup comparison. Exclusion criteria included individuals with motor, neurological, or visual impairments, and those necessitating handrails or assistance while descending stairs. Ultimately, 82 older adults (age: 72.1 ± 4.8 years; body height: 1.62 ± 0.09 m; body mass: 60.8 ± 11.9 kg) participated in the study. This sample size meets the requirements for cluster analysis and provides adequate statistical power for detecting differences among subgroups. Written informed consent was obtained from all participants after explaining the procedures and possible risks. The study was approved by the Kansai Medical University Ethics Committee (# 2020294) and conducted in accordance with the Declaration of Helsinki.

### Health status

Health status was evaluated utilizing the Charlson Comorbidity Index (CCI) [16], Montreal Cognitive Assessment-Japanese version (MoCA-J) [17], and Frenchay Activities Index (FAI) to assess the severity of concurrent medical conditions, cognitive functionality, and well-being based on the Instrumental Activities of Daily Living (IADL) [18].

### Motion analysis

We used an optical 3D motion capture system (Locus 3D MA-3000, Anima Corp., Tokyo, Japan) comprised of 12 infrared cameras with a sampling rate of 100 Hz, and two force plates (120 cm long x 60 cm wide) with a sampling rate of 1000 Hz (MG-1190, Anima Corp., Tokyo, Japan) installed on the right and left sides to enable synchronized measurement. The measurement error of the reflective markers was within 0.1% of the visual field, ensuring a high-level of accuracy. The intraclass correlation coefficients (ICC [1]) of the critical kinematic and kinetic data of the stair descent task used in this study were in the range of 0.84–0.95, indicating high reproducibility. After data acquisition, motion analysis was conducted utilizing the 3D motion analysis system’s software. A single step with vertical axis of 20 cm, anterior-posterior axis of 40.5 cm, and medio-lateral axis of 58 cm was installed in the posterior half of each force plate. Based on a previous study [19], twenty-six retroreflective markers (20 mm diameter) were placed bilaterally on the acromion, lateral humeral epicondyle, radial styloid process, anterior superior iliac spine, posterior superior iliac spine, greater trochanter, medial and lateral femoral epicondyle, medial and lateral malleolus, first and fifth metatarsal heads, and calcaneal tuberosity.

A forward step-down task, utilized in previous studies as a surrogate task for stair descent, was used given its incorporation of weight-bearing stress and muscular control [20, 21]. Initially, participants placed their feet on the corresponding steps of the staircase, maintained an upright stance while looking straight ahead, standing with their natural stance width, and evenly distributing their weight on both sides. Step descent was performed in three trials at a self-selected pace and step sequence without the use of handrails, with participants wearing harnesses for safety. Three trials were chosen to minimize the potential effects of habituation and fatigue while maintaining reliable data collection [22].

Marker trajectories and ground reaction forces (GRF) were filtered using a second-order Butterworth low-pass filter at 10 Hz and 20 Hz, respectively. Kinematic and kinetic data derived from the marker trajectories and GRF were adjusted and output at 100 Hz. Foot-strike and foot-off events were determined based on the vertical component of the GRF being above or below 10 N [23]. The stair-descent cycle was divided into five phases from the GRF signals of foot-strike and foot-off on both sides: leg off, controlled lowering (CL), weight acceptance (WA), leg pull through, and double support [24]. The CL phase demands considerable energy dissipation, requiring approximately three times the knee joint extension moment of walking on level ground [25]. Furthermore, quadriceps activity has been shown to play a crucial role in controlling CoM acceleration during the late stance phase of stair descent [26]. Therefore, kinematic and kinetic data in the CL phase should imply differences in muscle strength and balance among participants. Moreover, if the mechanical demands of the CL phase cannot be satisfied, the WA of the subsequent leading limb (L-limb) is likely to be affected. Consequently, this study scrutinized kinematic and kinetic data at the CL phase for the trailing limb (T-limb) and the WA phase for the L-limb.

Each joint angle during stair descent was calculated from the adjacent joint center positions, as recommended by the International Society for Biomechanics, to define the joint coordinate system in Euler angles [27]. Furthermore, the joint angles were corrected by subtracting each participant’s joint angle recorded in their upright standing position with evenly distributed weight prior to stair descent. The trunk progression angle was calculated by the angle between the line connecting the bilateral acromion and the medial-lateral axis of the staircase. The foot progression angle was calculated as the angle between forward progression and the line connecting the midpoints of the medial and lateral malleolus and the first and fifth metatarsal heads. We extracted data at the end of the CL phase on the T-limb side from these angle measurements during the stair cycle. The end of the CL phase on the T-limb corresponds to the beginning of the WA phase on the L-limb, which is indicated by GRF onset on the L-limb. Since all data were output at 10-millisecond intervals, data corresponding to the endpoint of the T-limb’s CL phase was derived from information collected at 10 milliseconds preceding GRF onset on the L-limb side. The external joint moments of the foot, knee, hip, and trunk were calculated using inverse dynamics based on the segment’s local coordinate system. Data extraction corresponding to the endpoint of the CL phase on the T-limb side was conducted synchronously with the joint angle, and the data corresponding to the endpoint of the WA phase on the L-limb side was extracted using GRF offset on the T-limb side.

CoM acceleration and the separation of CoM and center of pressure (CoP) are known risk factors for stair falls [9]. We also estimated these metrics as follows. The position of whole-body CoM was estimated as the weighted sum of the various body segments [28]. The trajectory of CoM during descent was smoothed with a 50-millisecond moving average for each vertical and anteroposterior component, based on previous studies [29, 30]. Afterward, CoM acceleration was calculated from the temporal change of the spatial position of the CoM, and the values at the endpoints of the CL phase on the T-limb and WA phase on the L-limb were extracted. CoM–CoP separation was calculated by projecting the positions of the CoM and CoP onto the force plate’s anteroposterior axis and measuring the distance between these points at the end of the CL phase on the T-limb side.

### Physical function

Physical function was measured by bilateral lower limb strength (hip extension, abduction, knee extension, and ankle plantar flexion), maximal walking velocity, the Timed Up and Go Test (TUG), and the Community Balance and Mobility Scale (CB&M).

Lower extremity muscle strength was measured using a belt-type dynamometer (µ-tus F1, Anima Corp., Tokyo, Japan) at the maximum isometric contraction. Muscle strength was normalized by multiplying the lever arm and dividing it by body weight in kg (Nm/BW). Knee extensor muscle strength was determined using a cutoff of 0.40 kg/BW for men and 0.31 kg/BW for women; below these values, hindrance to daily activities was observed [31]. The proportion of positive subjects in each cluster was calculated.

Participants’ maximum walking speed was determined by timing them while walking as fast as possible on a flat floor in a straight line for 10 m. The assessment of balance and mobility was conducted using TUG, which is widely used for predicting falls in community-dwelling older adults [32]. TUG measures the number of seconds it takes for a participant to stand up from a chair, walk three meters, turn around, walk back to the chair, and sit down [33].

This study also used CB&M, which can evaluate advanced balance and mobility while reducing the ceiling effect in community-dwelling older adults with high physical function [34, 35]. CB&M scores 13 tasks on a 6-point scale based on each criterion. Using factor analysis, Takacs et al. [36] demonstrated that CB&M comprises three elements: muscle strength, mobility, and standing balance ability. Thus, we also calculated the scores for each factor.

According to previous studies, the cutoff values for fall risk are less than 1.09 m/s for maximum walking speed [37], more than 13.5 s for TUG [32], and less than 59 points for CB&M [35]. We calculated the prevalence of fall-risk positive individuals in each cluster based on these values.

### Statistical analysis

JMP Pro (v16.2.0; SAS Institute, Cary, NC, USA) and G^*^power 3.1 were used for statistical analysis. A statistical significance of p < .05 was applied, and the post-hoc effect size for each statistical test was determined based on a comprehensive review [38].

Kinematic and kinetic data, as well as angle data used for cluster analysis, were averaged across three trials for each participant [25, 26]. To classify stair descent types, we conducted cluster analysis using a comprehensive angle dataset: Euler angles of the trunk, hip, knee, and ankle joints during the CL phase, and the trunk and foot progression angle. We determined the optimal number of clusters using the elbow method [39]. The constructed dendrogram was divided into the optimal number, and participants were assigned to the appropriate cluster.

A one-way ANOVA was conducted to compare age, height, weight, health status, and kinematic and kinetic data during stair descent among clusters. Post hoc analyses were performed using Tukey’s Honestly Significant Difference (HSD) test. Fisher’s exact test was employed to assess differences in ratios of sex, fall risk, and prevalence of muscle weakness during knee extension among the clusters. Residual analysis was conducted to identify the specific differences when Fisher’s exact test revealed a significant difference between clusters. The one-sample Wilcoxon test was utilized to compare the overall scores of CCI, MoCA-J, and FAI with the data from a previous studies involving Japanese community-dwelling elderly individuals [40–42].

## Results

### Phenotypic classification and participant characteristics

A hierarchical cluster analysis using Ward’s minimum variance method classified the descent phenotypes into three clusters: Cluster 1 (n = 20, 24.4%), Cluster 2 (n = 43, 52.4%), and Cluster 3 (n = 19, 23.2%).

Differences in demographic and clinical characteristics between the clusters are shown in Table 1.

**Table 1:** Demographic and clinical characteristics

	Cluster 1	Cluster 2	Cluster 3	Overall	P-value	Effect
	n = 20	n = 43	n = 19	n = 82		Size
Age (years)	71.4 ± 5.4	72.1 ± 4.8	72.8 ± 4.4	72.1 ± 4.8	0.65^*a*^	
Sex (F/M)	11/9	12/31	7/12	30/52	0.13^*b*^	
Height (m)	1.61 ± 0.11	1.63 ± 0.08	1.63 ± 0.09	1.62 ± 0.09	0.62^*a*^	
Weight (kg)	57.2 ± 12.7	62.2 ± 11.3	61.6 ± 12.1	60.8 ± 11.9	0.29^*a*^	
BMI	21.9 ± 2.5	23.4 ± 3.3	23.2 ± 3.4	23.0 ± 3.1	0.24^*a*^	
CCI	0.5 ± 0.9	0.8 ± 1.4	0.4 ± 1.0	0.6 ± 1.2	0.45^*a*^ < 0.05^*c*^	2.16^*c*^
MoCA-J	24.7 ± 0.6	24.3 ± 0.4	23.9 ± 0.6	24.3 ± 2.8	0.74^*a*^ 0.07^*c*^	
FAI	30.6 ± 6.0	30.3 ± 5.5	31.1 ± 6.0	30.6 ± 5.7	0.90^*a*^ < 0.05^*c*^	0.45^*c*^

Values are presented as mean ± standard deviation

*BMI* body mass index, *CCI *Charlson comorbidity index, *MoCA-J* montreal cognitive assessment-Japanese version, *FAI* Frenchay activities index

^*a*^Comparison between clusters using one-way ANOVA

^*b*^Comparison between clusters using Fisher’s exact test

^*c*^Comparison between participants overall and those of a previous study using the One-Sample Wilcoxon test

There were no significant differences in demographic and clinical characteristics among the three clusters. Compared with a prior study using the one-sample Wilcoxon test [40–42], the overall CCI scores of participants in this study were significantly lower, while FAI scores were significantly higher and the overall MoCA-J scores did not exhibit significant differences.

### Comparing kinematic and kinetic features between stair-descent clusters

Table 2 shows the kinematic differences between stair-descent clusters based on a one-way ANOVA and post hoc Tukey’s HSD test.

**Table 2:** Joint and progression angles of the trunk and trailing limb of the three clusters at the endpoint of the controlled lowering phase

A. Joint angle															
	Sagittal angles [°]	Cluster			Tukey HSD	Frontal angles [°]	Cluster			Tukey HSD	Horizontal angles [°]	Cluster			Tukey HSD
		1	2	3	Effect size		1	2	3	Effect size		1	2	3	Effect size
Trunk	Flexion*	− 0.45	− 4.9	− 1.8	1, 3 >2	Ipsilateral	0.0	− 0.29	1.6	3 >1, 2	Ipsilateral	5.6	4.0	14.0	3 >1, 2
		(3.0)	(3.1)	(4.6)	0.56	Lean*	(2.1)	(1.6)	(2.6)	0.39	Rotation*	(3.5)	(4.0)	(4.3)	1.02
Hip	Flexion*	10.5	1.3	8.3	1, 3 >2	Abduction*	− 6.2	− 1.2	− 0.7	2, 3 >1	External	− 4.4	− 1.6	− 9.6	1, 2 >3
		(3.4)	(5.1)	(6.7)	0.79		(3.2)	(3.9)	(4.1)	0.59	Rotation*	(4.3)	(4.7)	(4.0)	0.72
Knee	Flexion*	53.0	47.8	49.9	1 >2	Abduction*	0.3	− 0.7	2.3	3 >2	External	0.28	− 0.4	− 4.6	1, 2 >3
		(7.6)	(6.1)	(7.9)	0.31		(4.2)	(4.2)	(5.1)	0.27	Rotation*	(5.6)	(6.9)	(5.0)	0.32
Ankle	Dorsal flexion	28.5	26.6	24.1		Inversion*	3.5	8.3	8.8	2, 3 >1	Abduction*	3.0	− 1.0	− 2.1	1 >2, 3
		(5.9)	(5.5)	(6.6)			(2.8)	(3.8)	(3.4)	0.61		(4.2)	(4.8)	(6.5)	0.37

B. Progression angle				
[°]	Cluster			Tukey HSD
	1	2	3	Effect size
Trunk progression angle*	7.9	5.8	20.0	3 >1, 2
	(5.9)	(5.9)	(6.1)	0.97
Foot progression angle*	12.6	15.1	22.2	3 >1, 2
	(8.1)	(6.2)	(10.4)	0.45

Values indicate the mean and standard deviation (in parentheses) of each joint angle

*statistical significance using a one-way ANOVA (p < .05)

**Fig. 1:**
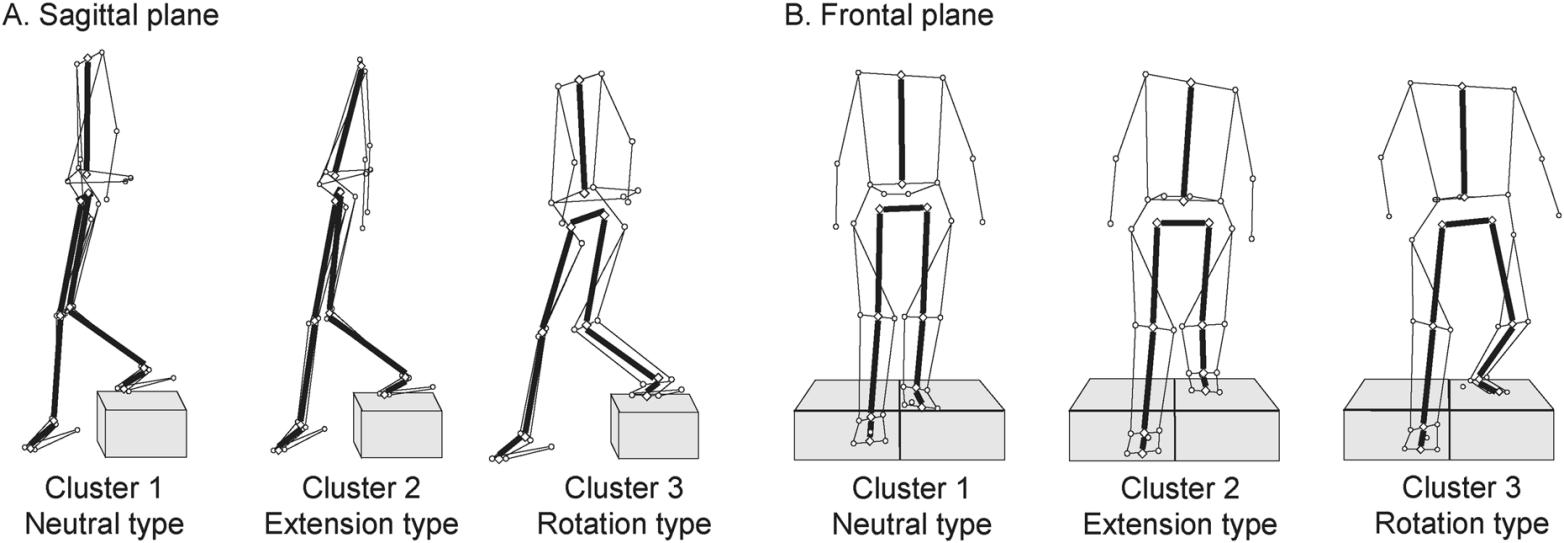
Specimen records in three clusters at the endpoint of the CL phase are shown in the sagittal (**A**) and frontal planes (**B**). Clusters 1–3 were termed based on their kinematic features during descent

In Cluster 2, there was a significant increase in trunk and hip extension during the CL phase, along with a decrease in knee flexion angle when compared to Cluster 1. Cluster 3 demonstrated a significant increase in the rotation angles of the trunk and lower extremities towards the stance side in comparison to Clusters 1 and 2. Additionally, the trunk and foot progression angles in Cluster 3 were oriented towards the stance side in comparison to Clusters 1 and 2. We observed substantial effect sizes, exceeding 0.4 [38], in the comparisons of sagittal and horizontal trunk and hip joint angles, as well as the progression angle between clusters. These alignment differences can be defined as distinctive characteristics of each cluster. Therefore, Cluster 2 is characterized by alignment on the sagittal plane and Cluster 3 is characterized by alignment on the horizontal plane and were thus named extension type (E-type) and rotation type (R-type), respectively. Cluster 1 maintains neutral alignment in the sagittal, frontal, and horizontal planes in comparison to Clusters 2 and 3 and was thus named neutral type (N-type; Fig. 1).

Table 3 shows the differences in joint moment, GRF vertical component, and mechanical stability between stair-descent clusters.

**Table 3:** Comparison of joint moment, GRF, and stability between the three clusters

A. Controlled lowering phase: trailing limb						B. Weight acceptance phase: leading limb					
Cluster		1	2	3	Tukey HSD	Cluster		1	2	3	Tukey HSD
		N-type	E-type	R-type	Effect size			N-type	E-type	R-type	Effect size
Joint moment [Nm/kg]						Joint moment [Nm/kg]					
Hip	Flexion*	0.14	0.33	0.10	2 >1, 3	Hip	Flexion*	0.19	0.12	0.21	
		(0.12)	(0.13)	(0.10)	0.88			(0.12)	(0.15)	(0.16)	
	Abduction*	0.31	0.26	0.14	1, 2 >3		Abduction*	0.39	0.43	0.36	
		(0.05)	(0.10)	(0.10)	0.67			(0.11)	(0.12)	(0.09)	
	Internal*	0.15	0.11	0.06			Internal	0.03	0.04	0.03	
	Rotation	(0.02)	(0.05)	(0.04)	0.78		Rotation	(0.02)	(0.04)	(0.04)	
Knee	Extension*	0.79	0.77	0.65	1, 2 >3	Knee	Extension*	0.09	0.21	0.12	2 >1
		(0.10)	(0.11)	(0.11)	0.49			(0.15)	(0.20)	(0.17)	0.30
	Abduction*	0.08	0.04	0.01	1 >2, 3		Abduction	0.16	0.19	0.17	
		(0.06)	(0.07)	(0.09)	0.35			(0.07)	(0.10)	(0.11)	
	Internal	0.04	0.02	0.04			Internal	0.03	0.04	0.02	
	Rotation	(0.04)	(0.05)	(0.05)			Rotation	(0.02)	(0.03)	(0.02)	
Ankle	Plantar	0.52	0.47	0.44	1 >2, 3	Ankle	Plantar	0.41	0.47	0.39	
	Flexion*	(0.06)	(0.08)	(0.08)	0.35		Flexion	(0.13)	(0.18)	(0.08)	
	Inversion*	0.02	0.01	0.04	3 >2		Inversion	0.03	0.03	0.03	
		(0.03)	(0.03)	(0.04)	0.40			(0.03)	(0.04)	(0.03)	
	Abduction	0.09	0.10	0.09			Abduction	0.04	0.06	0.05	
		(0.05)	(0.03)	(0.02)				(0.03)	(0.03)	(0.03)	
GRF [%BW]						GRF [%BW]					
Vertical*		87.3	81.7	76.6	1 >3	Vertical*		101.8	115.9	104.3	2 >1, 3
		(6.3)	(9.5)	(9.7)	0.42			(12.1)	(18.2)	(17.2)	0.39
Stability						Stability					
CoM acceleration		95.9	115.8	106.0		CoM deceleration		− 96.0	− 141.6	− 107.3	
Forward [cm/s^2^]		(38.9)	(33.5)	(35.3)		Forward [cm/s^2^]		(61.0)	(85.3)	(80.6)	
CoM acceleration		102.4	152.4	196.3	3 >1	CoM deceleration		− 50.1	− 203.3	− 95.3	1 >2
Vertical [cm/s^2^]*		(57.1)	(96.5)	(73.1)	0.38	Vertical [cm/s^2^]*		(131.4)	(201.1)	(174.0)	0.24
CoM-CoP		14.9	19.2	18.3	2, 3 >1						
Separation [cm]*		(3.2)	(3.7)	(3.8)	0.49						

Values indicate the mean and standard deviation (in parentheses)

*statistical significance according to a one-way ANOVA (p < .05)

*GRF* ground reaction force, *BW* body weight, *CoM* center of mass, *CoP* center of pressure

Regarding Cluster 2, hip flexion moment was larger than in Clusters 1 and 3, and ankle plantar flexion moment was significantly smaller than in Cluster 1 in the CL phase. Additionally, the CoM-CoP separation in the CL phase was significantly greater than that in Cluster 1, and the vertical GRF during the WA phase was significantly larger than that in Clusters 1 and 3.

Cluster 3 exhibited significantly smaller magnitudes of hip abduction, hip internal rotation, and knee extension moments during the CL phase compared to Clusters 1 and 2. The ankle plantar flexion moment was also smaller than in Cluster 1. Furthermore, during the CL phase, the vertical GRF in Cluster 3 was significantly lower compared to Cluster 1, accompanied by increased vertical CoM acceleration and increased CoM-CoP separation.

### Comparing balance function, mobility function, and lower limb strength between clusters

Table 4 shows the differences in lower limb strength, mobility function, and balance function among clusters, assessed using a one-way ANOVA and post hoc Tukey’s HSD test.

**Table 4:** Comparison of lower limb strength, balance function, and mobility function between three clusters

Cluster			1	2	3	Post-hoc	Effect
			N-type	E-type	R-type	Analysis	Size
Lower limb muscle strength							
Hip	Strength [Nm/kg]	T-limb	1.96 ± 0.56	1.84 ± 0.59	1.84 ± 0.58		
Extension		L-limb	1.94 ± 0.50	1.91 ± 0.59	1.79 ± 0.56		
Hip	Strength [Nm/kg]	T-limb	1.36 ± 0.33	1.34 ± 0.36	1.21 ± 0.40		
Abduction		L-limb	1.43 ± 0.36	1.28 ± 0.29	1.29 ± 0.38		
Knee	Strength [Nm/kg]	T-limb	2.20 ± 0.44	2.05 ± 0.52	1.98 ± 0.51		
Extension		L-limb	2.09 ± 0.44	2.11 ± 0.56	2.00 ± 0.59		
	Prevalence [%]		5.0	9.3	10.5		
Ankle	Strength [Nm/kg]	T-limb	2.23 ± 0.52	2.10 ± 0.49	2.01 ± 0.50		
Plantar flexion		L-limb	2.28 ± 0.58	2.09 ± 0.55	2.01 ± 0.58		
Balance and mobility function							
Walking	Speed [m/s]		2.2 ± 0.3	2.0 ± 0.3	2.0 ± 0.4		
	Prevalence [%]		0	0	0		
TUG	Time [s]		5.5 ± 0.5	6.1 ± 1.1	6.1 ± 0.9		
	Prevalence [%]		0	0	0		
CB&M	Total score*		80.5 ± 8.2	72.0 ± 12.2	68.5 ± 15.4	1 > 2, 3**	0.36
	Strength factor*		58.6 ± 5.9	50.4 ± 10.0	47.6 ± 12.3	1 > 2, 3**	0.41
	Balance factor*		17.1 ± 2.8	14.2 ± 4.8	13.0 ± 4.6	1 > 2, 3**	0.34
	Mobility factor		22.2 ± 3.9	22.1 ± 2.5	21.2 ± 4.9		
	Prevalence [%]†		0	18.6	26.3	2, 3 > 1^‡^	0.69

Values indicate the mean and standard deviation

*CB&M* community balance and mobility scale, *TUG* timed up and go test, *T-Limb *trailing limb, *L-limb* leading limb. *statistical significance using a one-way ANOVA (p < .05),

**statistical significance using Tukey’s HSD (p < .05),

† statistical significance using Fisher’s exact test (p < .05), ‡ statistical significance using residual analysis (p < .05)

Muscle strength related to hip extension, hip abduction, knee extension, and ankle plantar flexion did not differ significantly between clusters. Furthermore, there were no significant differences in walking speed or TUG.

Compared to Cluster 1, the CB&M total score was significantly lower in Clusters 2 and 3. Furthermore, the CB&M sub-analysis results indicated that Clusters 2 and 3 had significantly lower standing balance and muscle strength factors compared to Cluster 1, while there was no difference in mobility-related scores. None of the participants in any cluster were classified as having a positive fall risk based on walking speed or TUG values. However, among those with a positive fall risk based on their CB&M total score, Clusters 2 and 3 had more cases than Cluster 1, and residual analysis indicated that Cluster 1 had a significantly lower positivity rate.

## Discussion

### Phenotypic descent features

The differences in features between individual phenotypes are notably exhibited through upper body alignment. For example, the trunk of N-type participants descends while maintaining a nearly upright alignment on the frontal and sagittal planes. Among the three clusters, none of the 19 N-type participants were identified as having a positive fall risk based on the walking speed, TUG, and CB&M cutoff values. Only one N-type participant had a positive risk of daily activity limitation due to decreased knee extension strength. Therefore, N-types in our study possessed the most robust physical function among community-dwelling older adults.

Relative to N-type and R-type individuals, E-types exhibited unique alignment on the sagittal plane, showing greater extension of the trunk and hip joints, leading to a backward tilt of the upper body. R-types displayed a distinct kinematic pattern in the horizontal plane, characterized by rotation of their trunk and entire lower extremity towards the T-limb side during descent. R-types also demonstrated other unique kinematic characteristics, including an increased trunk and foot progression angle and lateral descent in a sideways stance while facing the T-limb side. Overall, we consider that N-types have a stable and low-risk descent pattern due to the absence of physical function abnormalities, whereas E-type and R-type descent patterns are likely to reflect a decline in physical function.

These findings support our hypothesis that stair-descent patterns in older adults can be classified into distinct phenotypes using 3D kinematic data. The three phenotypic differences observed reflect varying biomechanical strategies, likely influenced by differences in physical function such as strength and balance. This aligns with our expectation that older adults adopt specific kinematic patterns to compensate for functional deficits.

Hierarchical cluster analysis revealed that the walking patterns of independently living older adults can be classified into two types: one resembling the walking pattern of young, healthy individuals, and the other characterized by smaller hip joint angles in the sagittal plane [43]. Stair descent in older adults was classified into three distinct types, showing differences not only in hip joint movements but also in overall body motion. Since stair descent involves greater mechanical loads compared to level walking [25, 26], the increased number of types may reflect the use of more diverse strategies than those observed in level walking.

### Relationship between phenotypes and physical function

Total CB&M scores, which measures both balance and mobility, were found to be significantly lower for E-type and R-type individuals than for N-types. Although TUG is also designed to evaluate balance and mobility, no significant differences between clusters were detected. This could potentially be attributed to ceiling effects, whereby the TUG is unable to accurately discern disparities in balance ability among high-functioning older adults, as previously highlighted [34]. Differences in positive fall-risk rates between TUG and CB&M also imply the existence of a ceiling effect. Hence, E-type and R-type stair-descent patterns may be inadequate to accommodate the advanced balance and mobility capabilities that surpass the functions required by the TUG. These results further validate our hypothesis that physical factors such as balance ability, play a critical role in distinguishing stair-descent phenotypes.

The results of the CB&M sub-analysis indicate that factors related to balance and muscular strength are significantly lower in E-type and R-type individuals compared to N-types. However, there was no significant difference observed in maximal lower limb muscle strength. Nonetheless, factors related to mobility did not show significant differences among the types. Likewise, there were no significant differences observed between clusters regarding maximum walking speed. Thus, the kinematic characteristics of E-type and R-type individuals may be attributed to a potential weakness in advanced balance ability and the muscular control of posture.

### Phenotypic descent strategies

The E-type and R-type features during stair descent suggest either a manifestation of or compensation for poor balance ability. In E-types, the upper body is displaced backwards in the CL phase; there are two possible reasons for this. The first is an attempt to distance the body from height-related fears. When a person feels a fear of heights, their body bends backward displacing their CoP backward [44]. E-types with reduced balance ability may have extended their trunk hip joints because they were afraid of heights or falling despite the short descent. Another reason may be excessive forward movement of the CoM counteracted by backward movement of the upper body. CoM–CoP separation in the E-type was larger than that in N-type and R-type. This increased CoM–CoP distance implies a higher risk of losing CoM control during descent [45]. Trunk mass accounts for two-thirds of body mass, and CoM is strongly influenced by trunk movement [46]. Therefore, it is plausible that trunk and hip extension serve to counteract excessive anterior CoM movement and help reduce fall risk.

The R-type is characterized by rotation of the trunk and lower limbs towards the T-limb during the CL phase. This pattern may be a mechanism to reduce the muscular strength required for controlling stair descent. During the CL phase, the body undergoes progressive descent, primarily facilitated by the eccentric contraction of the ankle plantar flexors and knee extensors [24, 25]. In an experimental study involving intentional sideways descent in healthy older adults [47], a decrease in plantar flexor moments during the CL phase was observed, with no decrease in knee extensor moments. This suggests that a sideways descent strategy may offer a means of mitigating the mechanical load imposed on the plantar flexors. However, R-types in this study displayed not only reduced plantar flexion but also decreased knee extension moments, with an earlier release of GRF compared to N-types. As the study encouraged participants to descend naturally, it accurately reflects their physical function weaknesses in their descent patterns. Hence, the most likely reason for trunk and lower extremity rotation is reducing mechanical stress on the knee extensors and plantar flexors. Additionally, R-types exhibited greater CoM–CoP separation and vertical CoM acceleration during the CL phase compared to N-types, suggesting that R-type descending motion does not counteract fall risk. Overall, R-types seem to prioritize reducing mechanical stress on the plantar flexors and knee extensors over compensating for balance instability.

## Conclusion

We classified kinematic data relating to stair descent among community-dwelling older adults into three distinct phenotypes and examined their relationships with physical function. Hence, this study represents an inaugural effort to elucidate the relationship between stair-descent movement phenotypes and physical function.

### Clinical significance

Our findings suggest the need for advanced balance training and muscle strength exercises for E-type and R-type individuals to prevent falls. However, machine-based strength training did not alter the stair descent performance in older adults, suggesting that isolated strength training may not improve stability during stair descent [8]. Previous studies related to falls have shown that strength training alone is ineffective for fall prevention and recommend incorporating balance training into exercise programs [48]. Therefore, interventions designed to prevent falls during stair descent may also be more effective if they target both strength and balance such as Tai chi exercises [49]. Since the dynamic stability of stair descent relies on the knee extensors and ankle plantar flexor [7], activating these muscles during vertical movements while standing on one leg may be effective in preventing falls during stair descent among community-dwelling older adults.

### Limitations

1. This study’s participants exhibited a lower severity of comorbidities and higher IADL compared to previous studies in Japan. Therefore, the findings are limited to a subset of older adults who are relatively healthier and more active.
2. The single-step descent used in this study poses a lower level of difficulty compared to descending a typical flight of stairs, potentially resulting in less pronounced phenotypic characteristics.
3. While this study interprets phenotype variations from a physical functional perspective, the influence of psychological aspects, such as fear of falling, is also important to consider.
4. This study is observational in nature and cannot establish causal relationships between the biomechanical features of stair descent and physical function. While our findings highlight potential associations, further longitudinal or experimental studies are needed to confirm causality and elucidate the mechanisms underlying these relationships.
5. Many of the post-hoc effect sizes were large, but moderate ones were also included. Therefore, this study’s findings should be confirmed with larger datasets.

## Data Availability

The datasets recorded and analyzed during the study are available from the corresponding author on reasonable request.
